# Assaults against U.S. law enforcement officers in the line-of-duty: situational context and predictors of lethality

**DOI:** 10.1186/s40621-016-0094-3

**Published:** 2016-11-25

**Authors:** Cassandra K. Crifasi, Keshia M. Pollack, Daniel W. Webster

**Affiliations:** 1 Department of Health Policy and Management, Johns Hopkins Center for Gun Policy and Research, Johns Hopkins Bloomberg School of Public Health, 624 N. Broadway, Baltimore, MD 21205 USA; 2 Johns Hopkins Education and Research Center for Occupational Safety and Health, Johns Hopkins Bloomberg School of Public Health, 615 N. Wolfe St, Baltimore, MD 21205 USA; 3 Department of Health Policy and Management, Johns Hopkins Center for Injury Research and Policy, Johns Hopkins Bloomberg School of Public Health, 624 N. Broadway, Baltimore, MD 21205 USA

**Keywords:** Violence, Workplace, Law enforcement, Epidemiology

## Abstract

**Background:**

Research on occupational safety of law enforcement officers (LEOs) has primarily focused on fatal assaults. Nonfatal assaults, however, have received little attention. The goal of this study was to describe the situational contexts in which LEOs are assaulted, and compare these contexts and risks between fatal and nonfatal assaults in the U.S. Analyzing both types of assaults provides a more complete understanding of occupational safety and opportunities for intervention.

**Methods:**

This study includes a descriptive epidemiology of fatal and nonfatal assaults of LEOs in the U.S. and a pooled cross-sectional analysis of risk factors contributing to the odds of lethal assault. Data were collected from the Law Enforcement Officers Killed and Assaulted database. Descriptive statistics were used to characterize fatal and nonfatal assaults. Odds ratios were generated to understand the odds that an assault would result in a fatality.

**Results:**

Between 1998 and 2013, there were 791 fatal assaults and 2,022 nonfatal assaults of LEOs. Nearly 60% of primary wounds in fatal assaults were received to the head, neck, or throat while nearly 50% of primary wounds in nonfatal assaults were received to the arms/hands or below the waist. The odds that an assault resulted in a fatality decreased by 57% (OR 0.43, 95% CI 0.32 to 0.58) when a LEO was wearing body armor. LEOs experiencing an ambush or unprovoked attack had significantly increased odds of an assault resulting in a fatality (OR 3.27, 95% CI 1.83 to 5.85 and OR 2.24, 95% CI 1.44 to 3.47 respectively). LEOs that were disarmed during an encounter with a suspect had more than 2-fold increased odds of an assault resulting in a fatality (OR 2.24, 95% CI 1.48 to 3.38).

**Conclusions:**

There are specific situational and encounter characteristics that influence the lethality of an assault, which suggest strategies for prevention. Mandatory wear policies for the use of body armor could significantly reduce mortality among assaulted LEOs.

**Electronic supplementary material:**

The online version of this article (doi:10.1186/s40621-016-0094-3) contains supplementary material, which is available to authorized users.

## Background

Several characteristics of law enforcement as an occupation increase the risk of both fatal and nonfatal assault. Seven of the ten National Institute for Occupational Safety and Health’s risk factors for workplace violence (including assault) have been identified as pertinent for law enforcement officers (LEOs): contact with public; mobile work place; working with unstable or volatile people; working alone or in small numbers; working late at night or during early morning hours; working in high crime areas; and working in community based settings (Fridell et al. 2009).

LEOs in the U.S. experience a high rate of fatal occupational injuries (14.2/100,000) (Maguire et al. 2002). While this overall occupational fatality rate is comparable among other first responder populations (Maguire et al. 2002), 2014 data from the Bureau of Labor Statistics (BLS) shows an overall fatal occupational injury rate for U.S. workers of 3.3/100,000 (BLS 2015b). An importantly difference between LEO fatalities and other first responders is that a significant portion of LEO occupational fatalities are the result of injuries sustained during an assault by a suspect or criminal (Kaminski, Marvell 2002; Austin et al. 2015); the rate of occupational homicides among LEOs is 3 times higher than the national average (Tiesman et al. 2010). Fatal assaults of LEOs are contextual, situational, and often occur during the suspects’ efforts to escape after committing a crime (Margarita 1980). Prior research shows that when fatal LEO assaults occur, initiating traffic stops, investigating crimes, and interacting with potentially dangerous suspects are among the most common encounters scenarios (Hessl 2003; Tiesman et al. 2010). These incidents are more common during arrest situations and disturbance calls (Brandl 1996; Kercher et al. 2013; Swedler et al. 2014). LEOs often interact with suspects during or shortly after the commission of a crime and these suspects may attempt to evade capture by assaulting a LEO.

There are also a number of community-level factors that lead to increased risk of fatal assaults of LEOs. Areas with higher rates of crime (Peterson, Bailey 1988; Bailey, Peterson 1994; Lester 2001) and arrests (Fridell, Pate 2001) also see higher rates of LEO homicide. This elevated risk may be due to increased exposure to potential acts of violence while responding to calls and arresting suspects. Community-level poverty is also positively associated with risk of fatal LEO assault (Kyriacou et al. 2006) which may possibly be due to concentrated disadvantage and higher crime.

Since the 1980s, firearms have been the most common weapon used in the fatal assault of a LEO. Firearms consistently account for between 75 and 95% of the weapons used in LEO homicides (Tiesman et al. 2010; Brandl 1996; Kraus 1987; Margarita 1980; Kyriacou et al. 2006; Swedler et al. 2014; LaTourrette 2010). States with higher rates of gun ownership also see higher rates of LEO homicide (Swedler et al. 2015). The weapon used by the suspect is thought to be one of the biggest differences between fatal and nonfatal assaults involving LEOs. This concept is owed to the lethality of firearms and location of the wound (Fridell et al. 2009). Body armor has also been shown to influence the risk of an assault resulting in a fatality. The Federal Bureau of Investigation (FBI) and National Institute of Justice (NIJ), using a case-control design, found that LEOs not wearing armor were at 14 times greater risk of fatal injury (NIJ 2001).

Our study expands on previous work and explores the potential relationship between the lethality of the weapons used by suspects, location of the wound, and use of body armor and fatal assaults. Much of the research on assaults of LEOs to date has focused primarily on factors contributing to or studies of fatal or nonfatal assaults within single departments. Our particular research fills an important gap in the literature by describing the contexts in which LEOs are assaulted and comparing these contexts between fatal and nonfatal assaults. This comparison allows for the identification of factors that increase the odds that an assault will result in a fatality. Understanding these factors will offer better insight into the occupational safety risks LEOs face and allow for the development of interventions to address these risks.

## Methods

This study has two components. The first is a descriptive epidemiologic analysis to describe the distribution of assaults and assault characteristics among LEOs. The second is a pooled cross-sectional analysis comparing LEO demographics, and situational and encounter characteristics to predict which factors result in increased odds of lethal assaults.

### Data source for law enforcement officer assaults

Data for this study were assembled from the FBI’s Law Enforcement Officers Killed and Assaulted (LEOKA) database (FBI 2012). As part of the Uniform Crime Reporting program, the FBI generates this database from reports of every line-of-duty fatal assault (i.e., homicide), and nonfatal assault committed with a firearm or knife/cutting instrument that result in an injury (FBI 2004). Because the LEOKA database only captures nonfatal assaults committed with a knife/cutting instrument or firearm, this data represents only a subset of LEOs that experience a nonfatal assault. The database also includes LEOs that were off-duty if they were acting in an official capacity at the time of the assault (e.g., they identified themselves as an officer).

The LEOKA database contains a number of variables for each assault including those relevant to this study: suspects’ weapon type (e.g. firearm, blunt instrument, car), LEO assignment (e.g., one-officer vehicle), encounter (e.g., traffic stop, robbery in progress), location of the primary wound, distance from the suspect, whether the LEO fired his/her service weapon, and whether the LEO was wearing body armor when assaulted. Data for nonfatal assaults were not available in the LEOKA prior to 1998. Data for fatal assaults were available to the researchers back through 1984; however, this analysis only included fatal assaults from 1998 to 2013 to make appropriate comparisons between fatal and nonfatal assaults.

### Analytic methods

Descriptive statistics were used to describe differences in fatal and nonfatal assaults for encounter, assignment, primary wound location, use of body armor, and suspects’ weapon use. A pooled cross-sectional analysis was conducted to examine which factors (LEO demographics, and situational and encounter characteristics) were associated with the odds of a lethal outcome following an assault against a LEO. Assaults against LEOs were coded as those that resulted in a fatality compared to assaults that were not fatal. Simple logistic regression was used to calculate odds ratios (OR) for the factors hypothesized to be related to whether an assault would result in a fatality: 1) LEO characteristics-age, experience, race (measured as White, Black, Asian, Native American), use of body armor, being disarmed by the suspect, discharging of the service weapon; 2) situational characteristics-type of assignment, distance from the suspect, and type of weapon used by suspect; and 3) encounter characteristics-i.e., the type of call the LEO was on or responding to at the time of the assault (e.g., disturbance call, traffic stop, robbery in progress).

Multiple logistic regression (MLR) was used to evaluate which characteristics increased the odds of an assault resulting in a fatality while controlling for factors that simple logistic regression indicated was also associated with fatal outcomes. There were a number of variables that were significant in the single logistic regression model and considered for the multiple regression model. These variables were excluded from the multiple regression model if they became insignificant (*p* < 0.05), did not improve model fit (measured by AIC and BIC), or significantly inflated variance (see Additional file 1: Table S1 for the complete results from the simple logistic regression).

In the MLR model for odds of lethality, there was collinearity between a LEO’s age and his/her level of experience. There was also collinearity between whether a LEO was disarmed and assaulted with his/her own gun. Both age and whether a LEO was disarmed were retained in the multiple logistic regression. Age was retained, as LEOs are likely to have increasing experience as they age. Whether the LEO was disarmed was retained as he/she could not be assaulted with his/her own weapon if not first disarmed. The final model included assignment, encounter, primary wound location, suspects’ use of a firearm, the age of the LEO, and whether the LEO wore body armor, was disarmed, or fired his/her weapon.

Analyses were conducted using Stata IC v 13 (StataCorp 2013). This study was deemed to be “not human subjects” research by the Johns Hopkins Bloomberg School of Public Health Institutional Review Board.

## Results

### Descriptive epidemiology

Between 1998 and 2013, there were 791 fatal assaults and 2,022 nonfatal assaults of LEOs in the U.S. Descriptive statistics for LEO demographics, and situational and encounter characteristics are presented in Table 1. On average, LEOs that were fatally assaulted were slightly older and more experienced than those who experienced a nonfatal assault. There were no differences between fatal and nonfatal assaults by gender and race. Firearms were more commonly used in fatal assaults, with significant differences in the use of handguns and rifles between fatal and nonfatal assaults. Nonfatally assaulted LEOs were more likely to be wearing body armor and to have fired their weapons than those fatally assaulted.

**Table 1:** Descriptive statistics of fatal and nonfatal LEO assaults, 1998–2013

Characteristic	Fatal (*N =* 791) %	Nonfatal (*N =* 2,022) %
Mean Age (years)*	37.7	35.6
Average experience (months)*	127.3	118.3
Male	94	95
White	85	86
Firearm*	92	66
Handgun*	65	48
Rifle*	19	10
Shotgun	7	8
LEO Wearing Body Armor*	67	83
LEO Disarmed*	13	4
LEO Fired Weapon*	22	37
Primary Wound Location		
Head/Neck/Throat*	61	25
Upper Torso/Back*	30	18
Lower Torso/Back*	6	9
Below Waist*	2	19
Arms/Hands*	0	29
Assignment		
One-officer Vehicle	59	60
Two-officer Vehicle*	9	13
Detective	6	5
Off-duty*	9	4
Special Assignment	7	8
Undercover	3	3
Other	7	7
Encounter		
Investigative Activities*	14	18
Disturbance Call*	7	11
Domestic Call*	7	11
Attempting Other Arrest	11	11
Ambush*	8	2
Unprovoked Attack*	12	5
Burglary in Progress	2	2
Robbery in Progress	6	6
Tactical Situations*	7	10
Traffic Pursuits and Stops*	18	11
Drug-related	4	4
Handling Mentally Deranged Persons*	2	6
Handling/Transporting/Custody of Prisoners	3	3

*p < 0.05

There were statistically significant differences in the location of primary wound between fatal and nonfatal assaults. For fatal assaults, more than 60% of primary wounds were received to the head, neck, or throat, while nearly 50% of nonfatal primary wounds were received to the arms/hands or below the waist. The most common assignment for fatal and nonfatal assaults were one-officer vehicles assignments. Ambush, unprovoked attacks, and traffic stops and pursuits were statistically significantly more common encounters among fatally assaulted LEOs compared to those that experienced nonfatal assaults.

### Pooled cross-sectional analyses

Assaults that were committed with a firearm, when compared to other weapons used by suspects, significantly increased the odds of fatality (OR 4.37, 95% CI 3.10 to 6.10). If a LEO was wearing body armor at the time of the assault, the odds of that assault resulting in a fatality were reduced by 57% (OR 0.43, 95% CI 0.32 to 0.58). If a LEO was disarmed during the encounter (e.g., a LEO lost control of his/her gun during a struggle with the suspect), there was a more than two-fold increase in the odds that the assault would result in a fatality (OR 2.24, 95% CI 1.48 to 3.38). If a LEO discharged his/her weapon (regardless of whether the suspect was shot), the odds of that assault resulting in a fatality were 66% lower compared to LEOs that did not fire their weapon (OR 0.34, 95% CI 0.27 to 0.44) (Table 2).

**Table 2:** Multiple logistic regression estimates of odds ratios for lethal outcomes

Independent variable	OR^a^	95% CI^b^	*p*-value
Age of LEO	1.02**	1.00 to 1.03	<0.001
Suspect used Firearm	4.37**	3.10 to 6.10	<0.001
LEO Wearing Body Armor	0.43**	0.32 to 0.58	<0.001
LEO Disarmed	2.24**	1.48 to 3.38	<0.001
LEO Fired Weapon	0.34**	0.27 to 0.44	<0.001
Primary Wound (reference = head/neck/throat)			
Upper Torso/Back	0.68**	0.54 to 0.87	0.002
Lower Torso/Back	0.24**	0.17 to 0.36	<0.001
Below Waist	0.03**	0.02 to 0.06	<0.001
Assignment (reference = Two-officer Vehicle)			
One-officer Vehicle	1.45*	1.02 to 2.07	0.041
Detective	1.40	0.80 to 2.50	0.242
Off-duty	2.68**	1.50 to 4.81	0.001
Special Assignment	1.44	0.86 to 2.42	0.165
Undercover	1.18	0.59 to 2.39	0.641
Encounter (reference = Investigative Activities)			
Disturbance Call	0.99	0.63 to 1.55	0.948
Domestic Call	1.10	0.70 to 1.74	0.688
Attempting Other Arrest	1.46	0.96 to 2.21	0.076
Ambush	3.27**	1.83 to 5.85	<0.001
Unprovoked Attack	2.24**	1.44 to 3.47	<0.001
Burglary in Progress	1.06	0.48 to 2.36	0.887
Robbery in Progress	1.45	0.89 to 3.38	0.139
Tactical Situations	1.12	0.70 to 1.79	0.647
Traffic Pursuits and Stops	2.38**	1.64 to 3.46	<0.001
Drug-related	1.75	0.90 to 3.40	0.097
Handling Mentally Deranged Persons	0.56	0.27 to 01.16	0.121
Handling/Transporting/Custody of Prisoners	0.95	0.45 to 2.00	0.887

^a^Odds ratio

^b^Confidence interval

*p < 0.05, **p < 0.001

For LEOs assigned to a one-officer vehicle, the odds of a fatality were 45% higher than for LEOs assigned to a two-officer vehicle (OR 1.45, 95% CI 1.02 to 2.07). LEOs that were off-duty at the time of the assault had more than two-fold increased odds of an assault resulting in a fatality compared to LEOs on two-officer vehicle assignments (OR 2.68, 95% CI 1.50 to 4.81). A number of encounters increased the odds of fatal assaults: 238% increase for LEOs conducting “traffic pursuits and stops” (OR 2.38, 95% CI 1.64 to 3.46); 224% increase for LEOs experiencing an “unprovoked attack” (OR 2.24, 95% CI 1.44 to 2.47); and 327% increase for LEOs experiencing an “ambush” (OR 3.27, 95% CI 1.83 to 5.85) compared to LEOs conducting “investigative activities” (Table 2).

## Discussion

There have been a number of studies describing circumstances of LEO homicides, but very few have focused on nonfatal assaults. When considering the total burden of injuries in a worker population, fatalities are only one small component; for each fatality, there are many more nonfatal injuries (BLS 2015a). Exploring the similarities and differences between fatal and nonfatal LEO assaults and the characteristics of assaults that are common among fatalities is a necessary step toward understanding occupational safety among law enforcement. Understanding the characteristics and determinants of lethal encounters is important to creating a clear and complete picture of LEO safety, which can then guide prevention efforts to reduce these assaults.

The use of body armor was one key difference between fatal and nonfatal assaults. In particular, over 80% of LEOs experiencing a nonfatal assault were wearing body armor compared to less than 70% of LEOs experiencing a fatal assault. The findings indicate that wearing armor reduced the odds of an assault resulting in a fatality by 57%. An NIJ study of LEOs’ use of body armor found that while an estimated 93% of LEOs work in a department where body armor use is required, only 88% wearing their body armor all of the time (NIJ 2012). The decision to wear body armor, regardless of department policy, could be an indicator of attitudes toward risk reduction at the individual level. This potential indicator is an area of research that warrants additional attention. Departments that do not currently have mandatory wear policies, or those without clear written policies, should implement policies that require LEOs to wear armor at all time while on duty, which could significantly reduce LEO mortality resulting from assault.

Both ambush and unprovoked attack encounters more than doubled the odds that an assault would result in a fatality. It is conceivable that in these types of encounters LEOs may be caught off guard or have little time to respond or defend themselves. These factors may increase the likelihood of seeing these types of attacks result in fatalities more often. Additionally, we are seeing an increasing number of ambush and/or unprovoked attacks of LEOs. According to the National Law Enforcement Officers Memorial Fund (NLEOMF) 2016 Mid-Year Law Enforcement Officer Fatalities report, of the 32 fatal shootings of LEOs nearly 44% are the result of an ambush-style attack (NLEOMF 2016b), up from 14% in 2015 (NLEOMF 2016a). Data from our study indicates that, since 1998, 2016 has the highest proportion of LEO fatalities resulting from an ambush or unprovoked attack-the previous high was 32% in 2009. An interesting and urgent priority for future research is to identify what is driving this increase in fatal ambush and unprovoked attacks.

Previous research found about 10% of LEO homicides were committed with the LEOs own service weapon after the LEO was disarmed (Swedler et al. 2014). The findings of this study further illustrate this risk as they show that when a LEO is disarmed there is a more than 2-fold increase in the odds of an assault resulting in a fatality. A survey of Police Chiefs’ perceptions on a variety of issues related to firearms and firearm policy indicated very little support for requiring new guns be personalized (<28%) in the general population (Thompson et al. 2006). Research is needed to explore the acceptability of personalized guns for law enforcement agencies and whether the introduction of this technology would significantly influence risk of mortality among law enforcement.

There is currently much attention on use-of-force by law enforcement as well as when deadly force is or is not appropriate. The findings of this study indicate that when a LEO fires his/her weapon, the odds of an assault resulting in a fatality are decreased by 67%. While this data does not provide a counter-factual, in dangerous situations, it is possible that a LEO must use deadly force-or at least fire his/her service weapon-to avoid being killed. An important area of future research will be to construct timelines of assaults in which a LEO fired his/her service weapon to determine A) if force was necessary and appropriate; and B) whether the suspect or the LEO fired first.

There are a few limitations of this research related to the data source. The FBI has a strict definition of line-of-duty homicides. LEOs must be on-duty at the time of the assault, or off-duty but performing actions as though on-duty. This definition has the potential to miss or undercount fatal assaults compared to other data sources (Tiesman et al. 2013) and potentially nonfatal assaults. However, whenever the FBI is alerted that a LEO has died in the line-of-duty, the LEOKA program office works to gather relevant information about the incident rather than waiting for the agency to report. This reduces the likelihood that reporting LEO fatalities could be influenced by general reporting to the Uniform Crime Reporting system. The database also only collects information on nonfatal assaults committed with a firearm or knife/cutting instrument that result in an injury. Therefore, assaults committed with other weapons (e.g., blunt objects or fists) that result in injury would not be captured. There is also the possibility that the reporting of nonfatal assaults is restricted to the most serious injuries. This potential misclassification bias could make nonfatal assaults appear more similar to those that result in a fatality, and thus mask potential differences. Finally, these data only represent LEOs that were assaulted; thus, these data are not generalizable to LEOs that are not assaulted.

## Conclusion

This study is the first to our knowledge that compares the contexts of fatal and nonfatal assaults nationally and identifies specific situational and encounter characteristics that influence the lethality of an assault. This study fills a significant gap in understanding the occupational safety of LEOs and identifies areas of intervention and guidance for prevention such as increasing the use of body armor and areas of importance for future research.

## Additional file

Additional file 1: Supplemental Table. Simple logistic regression estimates of odds ratios for lethal outcomes. (DOCX 14 kb)
