# Supplementary material for: Assaults against U.S. law enforcement officers in the line-of-duty: situational context and predictors of lethality
Source: Inj Epidemiol. 2016 Nov 25;3:29. doi: 10.1186/s40621-016-0094-3 (PMC5122525; doi:10.1186/s40621-016-0094-3)
Supplement: Additional file 1: Supplemental Table. — Simple logistic regression estimates of odds ratios for lethal outcomes. (DOCX 14 kb) [file 40621_2016_94_MOESM1_ESM.docx]

Supplemental Table

| **Independent Variable** | **OR^a^** | **95% CI^b^** | **p-value** |
| --- | --- | --- | --- |
| Age of LEO | 1.03* | 1.02 to 1.04 | < 0.001 |
| Experience of LEO | 1.001* | 1.00 to 1.002 | 0.017 |
| Female LEO | 1.280 | 0.86 to 1.85 | 0.189 |
| LEO Wearing Body Armor | 0.42* | 0.34 to 0.50 | < 0.001 |
| LEO Disarmed | 3.51* | 2.59 to 4.76 | < 0.001 |
| LEO's Own Weapon Used by Suspect | 2.55* | 1.77 to 3.68 | < 0.001 |
| LEO Fired Weapon | 0.48* | 0.39 to 0.58 | < 0.001 |
| Distance from Suspect > 10 Feet | 0.84 | 0.67 to 1.03 | 0.102 |
| Type of Firearm Used (reference = handgun) | | | |
| Rifle | 1.44 | 1.14 to 1.83 | 0.003 |
| Shotgun | 0.64 | 0.46 to 0.88 | 0.005 |
| Caliber/Type of Firearm (reference = small/medium caliber handguns) | | | |
| Large Caliber Handgun | 1.29* | 1.03 to 1.62 | 0.024 |
| Rifle | 1.52* | 1.18 to 1.97 | 0.001 |
| Shotgun | 0.67* | 0.48 to 0.94 | 0.019 |
| Primary Wound (reference = head/neck/throat) |  |  |  |
| Upper Torso/Back | 0.71* | 0.58 to 0.87 | < 0.001 |
| Lower Torso/Back | 0.29* | 0.21 to 0.41 | < 0.001 |
| Below Waist | 0.04* | 0.02 to 0.06 | < 0.001 |
| Race (reference = white) |  |  |  |
| Black | 1.38* | 1.06 to 1.78 | 0.015 |
| Asian | 0.58 | 0.28 to 1.21 | 0.146 |
| Indian | 1.29 | 0.55 to 3.04 | 0.553 |
| Assignment (reference = Two-officer Vehicle) | | | |
| One-officer Vehicle | 1.36* | 1.03 to 1.80 | 0.033 |
| Detective | 1.68* | 1.10 to 2.78 | 0.016 |
| Off-duty | 3.25* | 2.14 to 4.94 | < 0.001 |
| Special Assignment | 1.34 | 0.90 to 2.00 | 0.150 |
| Undercover | 1.33 | 0.79 to 2.24 | 0.281 |
| Other LEOs Assisted (reference = Assisted) | | | |
| Alone, Assistance Requested | 1.15 | 0.91 to 1.45 | 0.229 |
| Alone, No Assistance Requested | 3.59* | 2.87 to 4.50 | < 0.001 |
| Encounter (reference = Investigative Activities) | | | |
| Disturbance Call | 0.78 | 0.55 to 1.13 | 0.192 |
| Domestic Call | 0.80 | 0.56 to 1.15 | 0.235 |
| Attempting Other Arrest | 1.23 | 0.88 to 1.70 | 0.223 |
| Ambush | 6.08* | 3.78 to 9.77 | < 0.001 |
| Unprovoked Attack | 3.24* | 2.28 to 4.60 | < 0.001 |
| Burglary in Progress | 0.95 | 0.51 to 1.79 | 0.886 |
| Robbery in Progress | 1.46 | 0.99 to 2.15 | 0.059 |
| Tactical Situations | 0.97 | 0.67 to 1.40 | 0.869 |
| Traffic Pursuits and Stops | 2.27* | 1.68 to 3.06 | < 0.001 |
| Drug-related | 1.32 | 0.81 to 2.14 | 0.265 |
| Handling Mentally Deranged Persons | 0.33* | 0.18 to 0.61 | < 0.001 |
| Handling/Transporting/Custody of Prisoners | 1.36 | 0.79 to 2.33 | 0.262 |
| ^a^Odds Ratio |  |  |  |
| ^b^Confidence Interval |  |  |  |
| * p < 0.05 |  |  |  |
